# Inverse PCR-based detection reveal novel mobile genetic elements and their associated genes in the human oral metagenome

**DOI:** 10.1186/s12903-022-02209-y

**Published:** 2022-05-27

**Authors:** Supathep Tansirichaiya, Endre Winje, Johannes Wigand, Mohammed Al-Haroni

**Affiliations:** 1 Department of Microbiology, Faculty of Medicine Siriraj Hospital, Mahidol University, Bangkok, Thailand; 2 Department of Clinical Dentistry, Faculty of Health Sciences, UiT the Arctic University of Norway, 9037 Tromsø, Norway; 3 Centre for New Antimicrobial Strategies, UiT the Arctic University of Norway, Tromsø, Norway

**Keywords:** Oral metagenome, Inverse PCR, Mobile genetic elements, Composite transposons, Tn*916* conjugative transposons, Integrons

## Abstract

The human oral cavity is one of the hotspots harboring multiple mobile genetic elements (MGEs), which are segments of DNA that can move either within bacterial genomes or between bacterial cells that can facilitate the spreading of genetic materials, including antimicrobial resistance genes. It is, therefore, important to investigate genes associated with the MGEs as they have a high probability of dissemination within the bacterial population under selective pressure from human activities. As one-third of oral bacteria are not yet culturable in the laboratory condition, therefore, in this work, it is aimed to detect and identify the genetic contexts of MGEs in the oral cavity through an inverse PCR (IPCR)-based approach on the oral metagenomic. The human oral metagenome was extracted from saliva samples collected from healthy individuals in Tromsø, Norway. The extracted DNA was partially digested with the HindIII restriction enzyme and self-circularized by ligation. DNA primers targeting each MGE were designed to amplify outwards from the MGEs and used for the IPCR on the circularized DNA products. The IPCR amplicons were cloned into a pCR-XL-2-TOP vector, screened, and sequenced. Out of 40 IPCR amplicons, we confirmed and verified the genetic contexts of 11 samples amplified with primers targeting integron gene cassettes (GCs), IS*431* composite transposons, and Tn*916* conjugative transposons (*tet*(M) and *xis*-*int*). Novel integron GCs, MGEs, and variants of Tn*916* conjugative transposons were identified, which is the first report using the IPCR technique to detect the genetic contexts of MGEs in the oral metagenomic DNA.

**Supplementary Information:**

The online version contains supplementary material available at 10.1186/s12903-022-02209-y.

## Introduction

The human oral cavity is the second most complex microbial ecosystem in the human body, inhabited by a comprehensive bacterial flora consisting of more than 700 bacterial species, of which 100–200 usually varies between individuals [60, 62]. The oral cavity has dynamic physio-chemical conditions as it is a major gateway to the human body, so the oral bacteria have to adapt to these changes from food, drink, air, and human-related behaviors such as brushing, smoking, and kissing [25, 28, 49]. Because of the high clearance rate of planktonic bacteria caused by the continuous flow of saliva in the oral cavity, most bacteria live as a structured community inside a matrix of extracellular polysaccharides (EPS) of a biofilm called dental plaque [5, 23]. Biofilm is a well-known virulence factor because of its protective and sustaining nature towards the inhabiting microbes.

Biofilm was also shown to facilitate a process called horizontal gene transfer (HGT) that bacteria use to exchange their genetic materials, including antimicrobial resistance genes (ARGs) [24, 30, 45]. There are three main types of HGT, including transformation, conjugation, and transduction. For example, a conjugative transposon Tn*5397*, conferring tetracycline resistance, was shown to be transferred from a nonoral *Bacillus subtilis* donor to an oral *Streptococcus* sp. in a mixed-species biofilm [46]. The dynamic change in the oral cavity also provides a selective pressure that drives HGT in the oral community, which is important for their adaptability and evolution, including the spreading of ARGs among the oral commensal and possibly to other pathogens.

HGT has been shown to be facilitated by mobile genetic elements (MGEs), which are segments of DNA that inherit the ability to translocate from one bacterial replicon to another. It could facilitate the transfer either within a bacterial cell or between two different cells. Integrative conjugative elements, including conjugative plasmids and conjugative transposons, can facilitate intercellular transfer through conjugation. Even though the other MGEs, such as insertion sequences (ISs), composite transposons, unit transposons, and integrons, could not facilitate the transfer between cells by themselves. They can move within the bacterial cell, inserting themselves onto ICEs for an intercellular transfer. IS elements are the simplest MGE, containing only gene(s) for their transposition [51]. Other MGEs can carry additional or accessory genes, including ARGs, as part of their structure, such as DNA fragment between two IS elements of composite transposons and gene cassette (GC) array of integrons [38, 47, 54, 57, 58].

Genes associated with MGEs have a higher probability to be disseminated in the bacterial population when the MGEs move. Previously, the spread of ARGs against last-resort drugs was reported to be mediated by MGEs in several studies such as, *mcr-1* colistin resistance gene that can be mediated by various plasmid types (IncI2, IncHI2, and IncX4) and transposons (Tn*6330* and Tn*6390*) [32, 35, 53, 63]. For the oral cavity, several resistance genes have also been shown to be associated with MGEs, such as *tet(M)* tetracycline resistance genes in Tn*916*-family conjugative transposons, *ddl6* D-cycloserine resistance gene in an integron GC, *qrg* Cetyltrimethylammonium bromide (CTAB) resistance gene on an IS*1216* composite transposon, and *knt* kanamycin resistance gene on IS*257* composite transposon [6, 41, 44, 56, 59].

As one-third of the oral bacteria have not been cultured in the laboratory condition yet [11, 61], investigating of MGEs and their associated genes through a culture-independent method like metagenomics would be suitable as it would allow us to study these genes from an entire oral microbiome. Previously, genes associated with integrons and composite transposons in the oral metagenomic DNA have been studied through a PCR-based metagenomic approach [56, 56, 59, 59], [57, 58]. These studies amplified and identified the genetic context of both MGEs by designing DNA primers to amplify in the outward direction from common features flanking the DNA carried by the MGEs (insertion sequences (ISs) for composite transposons and *attC* recombination sites for integrons).

**Fig. 1:**
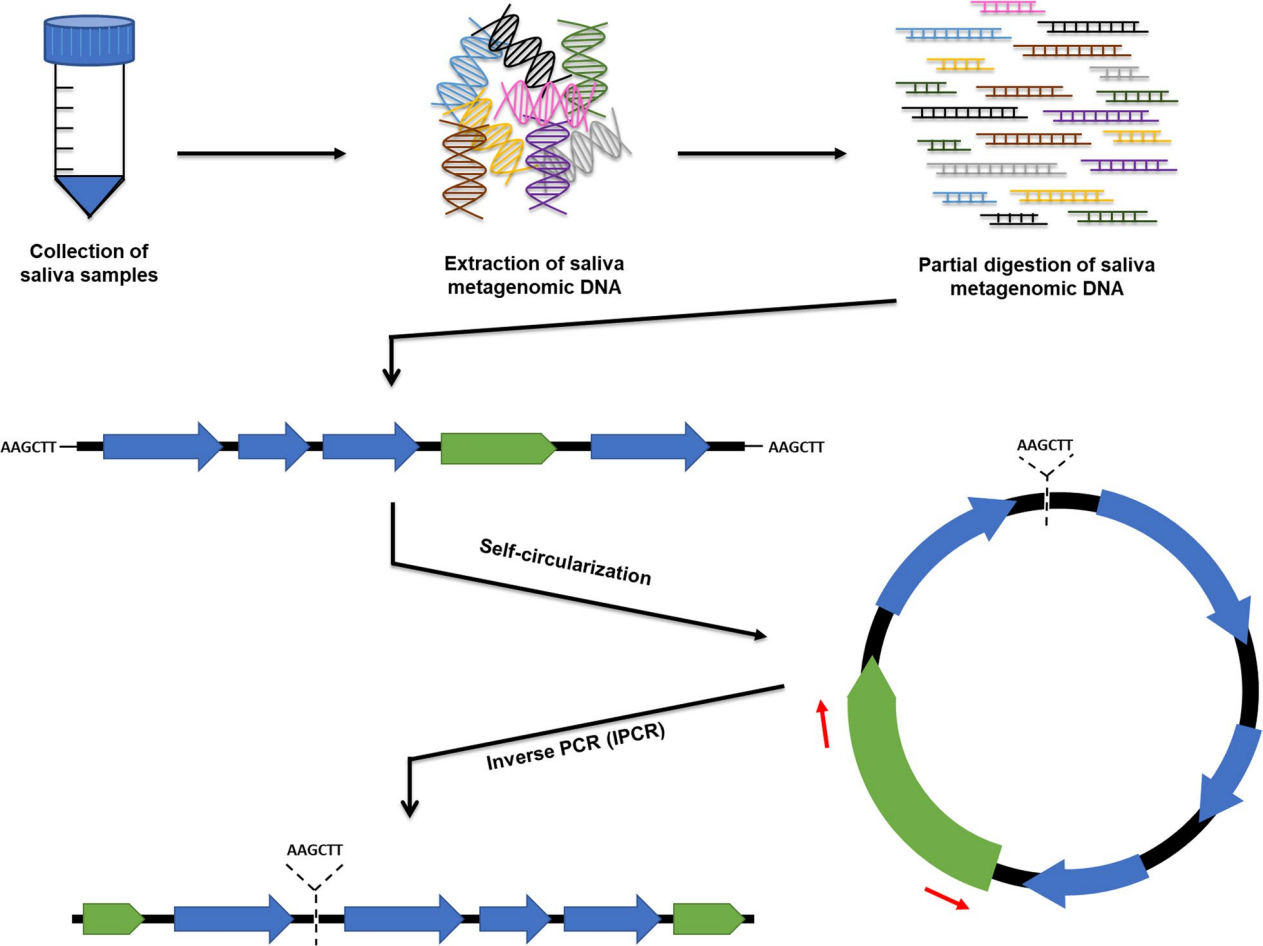
Schematic representation of IPCR-based approach to determine the genetic context of MGEs in the human oral metagenome. Saliva samples were collected from healthy individuals, and oral metagenomic DNA was subsequently extracted from each sample. The extracted metagenome was partially digested with HindIII restriction enzyme and self-ligated as circular DNA molecules to use as templates for IPCR. The unknown genetic context (blue open arrows) of each MGE (green arrows) can then be detected by designing DNA primers (red arrows) to amplify outwards from the MGE. The IPCR amplicons are cloned into the vector, sequenced, and analyzed by bioinformatics

However, not all MGEs have two common features flanking their cargo DNA, so their genetic context cannot be investigated by conventional PCR. An inverse PCR (IPCR) is a technique that could overcome this limitation as it involves digestion of original template DNA, self-ligation (circularization) of the digested products, and uses these self-circularized DNA molecules as templates for PCR (Fig. 1). Using IPCR on oral metagenomic DNA would therefore allow us to investigate and identify the genetic context upstream and downstream from a known single section of MGEs, which have a high probability to be disseminated in the oral microbiome. It was previously used to investigate the genetic context of resistance genes in sediment metagenome, which they found novel MGEs, and new putative ARGs [37].

In this study, we aimed to investigate the genetic context of MGEs in the human oral cavity through an IPCR-based metagenomic approach. The presence of MGEs in the Norwegian oral metagenome was confirmed by using both previously published primers and newly designed primers. Another set of primers amplifying outwards from the MGEs were then designed and used for the IPCR on the self-circularized oral metagenomic DNA template. Several novel integron GCs and variants of Tn*916* were identified.

## Materials and methods

### Study population, Saliva sample collection and extraction of oral metagenomic DNA

Saliva samples were collected from 50 healthy volunteers between 21 and 65, both male and female, who visited the University Dental Clinic at UiT the Arctic University of Norway. The inclusion criteria include not having been treated with antibiotics in the last 3 months and not having chronic diseases. Participants were asked to abstain from drinking, eating, and brushing their teeth at least one hour before the saliva collection. Ethical approval was obtained from the Regional Committees for Medical and Health Research Ethics (REK) prior to the sample collection (Project number 2018/1373/REK nord). All participants were properly informed and gave their written consent. A paraffin gum was used to stimulate saliva secretion, and approximately 2 mL of saliva was collected from each participant using Norgen’s Saliva DNA Collection, Preservation and Isolation Kit (Norgen Biotek Corp, Ontario, Canada). All the saliva samples were anonymized and stored at room temperature according to the manufacturer’s instructions.

### Extraction of the oral metagenomic DNA

750 µL of saliva from each sample was transferred to a 2-mL microcentrifuge tube and mixed with 750 µl of phosphate-buffered saline (PBS). Each tube was then centrifuged for 10 min at 15,700 ×*g* to pellet the cells. The supernatant was discarded and resuspended the pellet in 125 μl PBS buffer and 25 μl MetaPolyzyme (Sigma-Aldrich, Norway). After incubating the tubes at 35 °C for 4 h, oral metagenomic DNA was extracted from each saliva sample by QIAcube (Qiagen, Norway) and QIAamp**®** DNA Mini QIAcube Kit. A pooled metagenomic DNA sample was prepared by aliquot and mixing 10 µl of each extracted oral metagenomic DNA in a new Eppendorf tube and kept at − 20 °C.

### Confirmation of MGEs in oral metagenomic DNA

The presence of each MGE in oral metagenomic DNA was first determined by primers listed in Additional file 1: Table S1. Primers targeting plasmids, Tn*916*-family conjugative transposons, and integrons were selected from previously published studies that had been used to successfully amplify the MGEs based on their conserved genes/sequences. For composite transposons, a list of composite transposons associated with ARGs was selected from the Transposon Registry [57, 58]. Then, primers were designed to target the conserved regions of their IS elements, identified by aligning available gene sequences from the GenBank nucleotide database and ISFinder [52], using Clustal Omega and Primer3 web-based software (https://bioinfo.ut.ee/primer3-0.4.0/).

PCR reactions of each primer pair were set up, containing 1 µl of the pooled oral metagenomic DNA, 2 µl of each primer (10 µM), 15 µl of 2 × Biomix red (Bioline, Norway), and 10 µl molecular grade water. The PCR cycle was programmed as follows: (i) initial denaturation at 95 °C for 5 min, (ii.) denaturation at 95 °C for 1 min, (iii.) annealing at 50–65 °C depending on the primers for 30 s, (iv.) elongation at 72 °C for 1 min 30 s, repeated step (ii.)–(iv.) for 35 cycles, and finished with a final elongation at 72 °C for 5 min. Amplicons from each PCR were cleaned using QIAquick PCR Purification Kit (Qiagen, Norway), following the protocol from the manufacturer. The presence of MGEs was confirmed by checking for the expected band of each MGE on agarose gels and by Sanger sequencing of the amplicons at the Genewiz, Germany.

### Digestion and circularization of the oral metagenomic DNA

The pooled oral metagenomic DNA was partially digested with HindIII restriction enzyme at 37 °C for 2, 3, and 4 min. The digested products were run on an agarose gel, and DNA products larger than 1000 bp were cut out and extracted from the gel by using QIAgen gel extraction kit (QIAgen, Norway), following the instructions from the manufacturer. After the gel extraction, the DNA fragments were subjected to self-ligation and formed as circular forms by setting up ligation reaction as follows: 1 µl T4 DNA ligase (0.05 U/μL), 10 µl 10X T4 DNA ligase buffer, 50–100 ng of the digested DNA product and topped up with molecular grade water to a final volume of 100 µl. The self-ligation reaction was incubated at 16 °C overnight and purified by using QIAquick PCR Purification Kit, following the protocol from the manufacturer.

### Amplification and cloning of the genetic context of the MGEs in the oral metagenomic DNA

The genetic context of the detected MGEs in the human oral metagenome was determined through the uses of IPCR primers (listed in Additional file 1: Table S1), amplifying outwards from the MGE into the flanking areas to use for the IPCR, which were designed by reverse complementing the MGE confirmation primers. The IPCR reactions consisted of 25 µl Platinum SuperFi Green PCR Master Mix (ThermoFisher, Norway), 2.5 µl of each inverse-PCR primers (10 µM), 5–50 ng of the self-circularized metagenomic DNA, and top up with molecular grade water to a final volume of 50 µl. The PCR cycle was programmed as follows: (i.) initial denaturation at 98 °C for 5 min, (ii.) denaturation at 98 °C for 1 min, (iii.) annealing at 50–65 °C depending on the primers for 30 s, (iv.) elongation at 72 °C for 6 min, repeated step (ii.)–(iv.) for 35 cycles, and finished with a final elongation at 72 °C for 5 min.

The amplicons were cloned into pCR™-XL-2-TOPO™ vectors through TOPO cloning using the TOPO™ XL-2 Complete PCR Cloning Kit (ThermoFisher, Norway), which is suitable for the cloning of long PCR products (up to 13 kbp), by following the protocol from the manufacturer. The ligated product was then transformed into One Shot™ OmniMAX™ 2 T1R chemically competent *Escherichia coli* cells using a standard heat shock protocol. The transformants were grown on LB agar supplemented with ampicillin (100 µg/ml) and incubated overnight at 37 °C.

### Plasmid isolation and sequencing of the extracted plasmids

The transformants of each MGE were screened by performing colony PCR with T3 and T7 primers and checked the colony PCR products on agarose gels. Colonies with different insert sizes and larger than 1000 bp were selected and subcultured into 5 ml of LB broth supplemented with ampicillin (100 µg/ml), then incubated overnight in a 37 °C shaker (200 rpm). The extracted plasmids were isolated from the overnight culture by using QIAprep Spin Miniprep Kit (QIAgen, Norway). All plasmids were sequenced by Sanger sequencing service at the Genewiz, Germany, using M13 Forward and M13 Reverse as initial primers for sequencing. Additional primers were designed and used for additional sequencing for the samples with longer inserts by using Primer3 (https://bioinfo.ut.ee/primer3-0.4.0/).

### Bioinformatics analysis

The sequencing data was visualized and assembled with CAP contig function by using BioEdit software version 7.2.0 (http://www.mbio.ncsu.edu/bioedit/bioedit.html) [22]. The contamination of vector sequences was checked and removed by using the VecScreen analysis tool (https://www.ncbi.nlm.nih.gov/tools/vecscreen/). The presence of both forward and reverse primers, and the conserved sequences of each MGE were then identified by searching and comparing the sequences. For the integron gene cassette samples, additional criteria were applied, such as the presence of *attC* core sites, as described by previous studies [56–59]. The sequences were compared to the nucleotide and protein databases in GenBank with BlastN and BlastX, respectively [1]. All sequences detected in this study were deposited to the GenBank under accession numbers from OL695865 to OL695875.

## Results

### Confirmation of MGEs in oral metagenomic DNA

A collection of MGEs, known to be associated with ARGs, was selected to be investigated in this study, including 4 Inc-type plasmids (IncP-1α, IncP-1β, IncP-9, and IncQ), Tn*21*-related transposons, Tn*916*-family conjugative transposons, integrons, and 17 composite transposons. Prior to the investigation of the genetic context of MGEs by IPCR, the presence of each MGEs in oral metagenomic DNA was determined through PCR using previously published primers, except for composite transposons, which were detected by using new primers designed to target the IS elements of each composite transposons (Additional file 1: Table S1). Sequencing the amplicons of the expected size confirmed the presence of 3 plasmid types, integrons, Tn*21*-related transposons, Tn*916*-family conjugative transposons, and 8 out of 17 IS elements in the oral metagenomic DNA (Table 1).

**Table 1:** List of MGEs that were presence in the extracted oral metagenome

MGEs	Name/Types of MGEs
Plasmids	IncP-1α
	IncP-1β
	IncP-9
IS elements	IS*1*
	IS*26*
	IS*431*
	IS*1182*
	IS*1216*
	IS*4351*
	IS*6100*
	IS*Aba1*
Transposons	Tn*21*-related transposons
	Tn*916*-family conjugative transposons
Integrons	Integron gene cassette (*attC*)

### *Identification and characterization of genes associated with MGEs *via* IPCR*

Another set of primers was designed to amplify outward from the common features of the confirmed MGEs and used in the IPCR on the self-circularized oral metagenomic DNA. The amplicons from IPCR could be either the DNA region flanked by two repeats of MGEs (IS elements of composite transposons and *attC* recombination sites of integrons) or the upstream and downstream genetic contexts of a single conserved gene on MGEs.

**Fig. 2:**
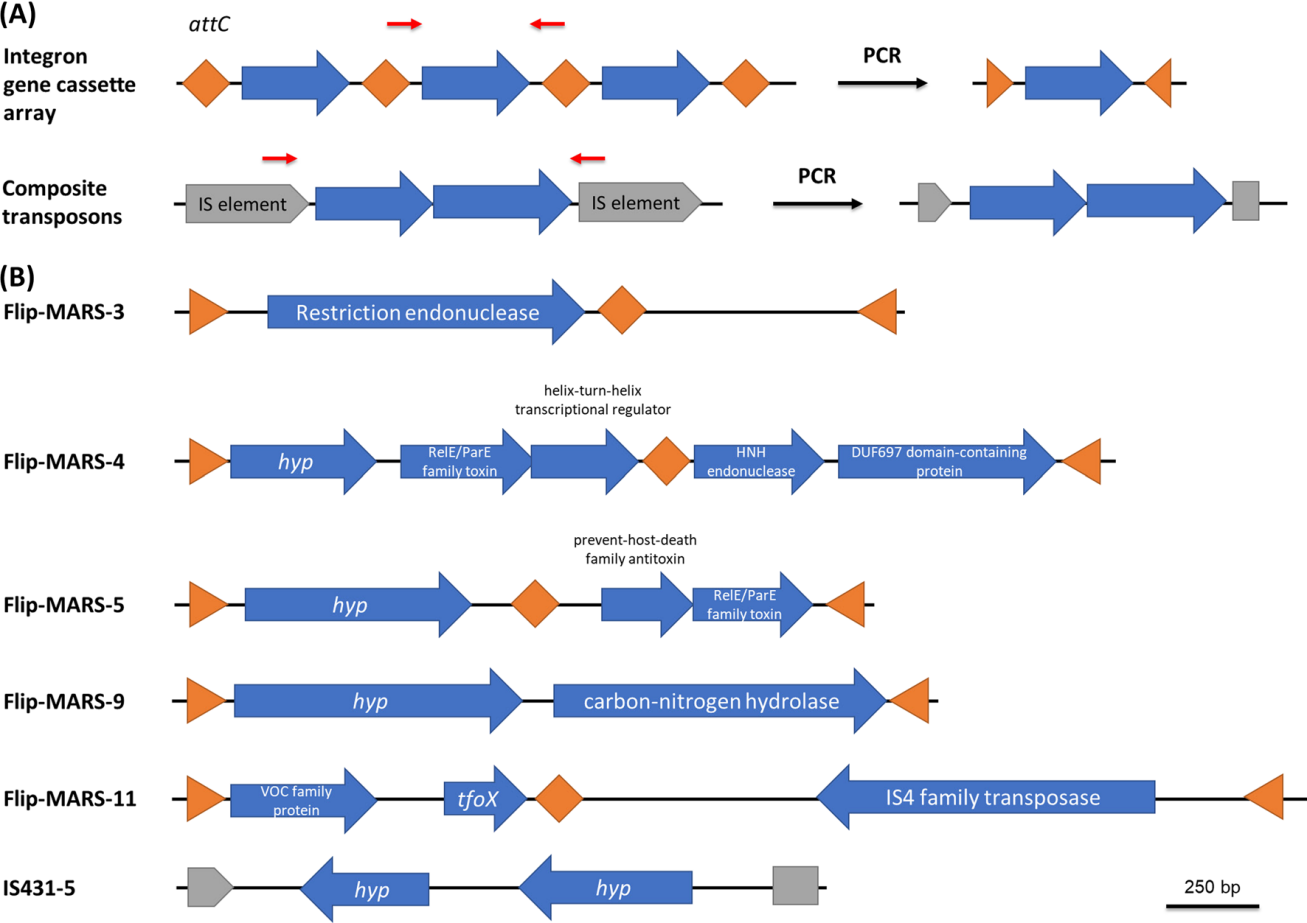
The predicted structures of the genetic context of integron GC and composite transposon samples detected from the oral metagenome. **A** The general structures of integron and composite transposons **B** The integron GC and composite transposons identified from the oral metagenome by IPCR. Genes associated with both types of MGEs were amplified from the DNA fragment (blue open arrows) located in between two DNA repeats which are *attC* recombination site of integrons (orange diamond shapes) and IS elements of composite transposons (grey open arrows)

Screening and bioinformatic analysis confirmed that 11 out of 40 IPCR DNA fragments as true amplicons, not PCR artifacts, with a size between 1.7 and 5 kb. The details and predicted genes on each IPCR amplicon are shown in Table 2. These samples could be divided into two groups. The first group was amplified from two DNA repeats, including 5 integron GC samples and 1 IS*431* composite transposon (Fig. 2). All integron gene cassettes were predicted to derive from *Treponema* species, and most of them, except Flip-MARS-9, contained two GCs. Several proteins were predicted to be encoded by these integron GC samples, such as toxin-antitoxin proteins (Flip-MARS-4 and Flip-MARS-5), endonuclease enzymes (Flip-MARS-3 and Flip-MARS-4), carbon–nitrogen hydrolase (Flip-MARS-9), vicinal oxygen chelate (VOC) family protein, and competence protein TfoX (Flip-MARS-11). Also, IS4 family transposase was identified in the Flip-MARS-11 sample, which was similar to the transposase on IS*Pca1* with 36% identities based on the BlastX analysis on ISFinder. For the IS431-5 composite transposon sample, it was predicted to contain two hypothetical proteins.

**Table 2:** Details of MGEs detected from the human oral metagenome by IPCR

Sample name (Accession number)	Primer pair	Size (bp)	BlastN				BlastX					
			Closest homologue	Percentage identity (%)	Coverage (%)	Accession number of the homologous DNA (BlastN)	Closest homologue	ORF size (bp)	Percentage identity (%)	Coverage (%)	Position on sample	Accession number of the homologous proteins (BlastX)
Flip-MARS-3 (OL695865)	MARS2-MARS5	1824	Uncultured bacterium clone MMU-PRO-6 gene cassette	91	38	MH536761.1	Restriction endonuclease [*Treponema* sp.]	852	55	99.6	211–1062	MBO6220054.1
Flip-MARS-4 (OL695866)	MARS2-MARS5	2355	*Treponema* sp. OMZ 804 chromosome, complete genome	91.9	42	CP048020.1	No significant similarity found	387	–	–	67–453	–
							Type II toxin-antitoxin system RelE/ParE family toxin [*Treponema succinifaciens*]	360	95	100	611–970	WP_162664812.1
							Helix-turn-helix transcriptional regulator [*Treponema* sp.]	288	96	100	960–1247	MBP5436565.1
							HNH endonuclease [*Treponema* sp. OMZ 804]	348	91	100	1357–1704	WP_162663704.1
							DUF697 domain-containing protein [*Treponema* sp. OMZ 804]	585	95	100	1744–2328	WP_162664812.1
Flip-MARS-5 (OL695867)	MARS2-MARS5	1704	*Treponema* sp. OMZ 838, complete genome	95	45	CP009227.1	Hypothetical protein [*Treponema pedis*]	609	91	100	116–724	WP_024468093.1
							Type II toxin-antitoxin system prevent-host-death family antitoxin [*Treponema*]	246	96	98.8	1110–1355	WP_002690260.1
							Type II toxin-antitoxin system RelE/ParE family toxin [*Treponema medium*]	321	95	100	1355–1675	WP_016522547.1
Flip-MARS-9 (OL695868)	MARS2-MARS5	1912	*Treponema denticola* ATCC 35,405, complete genome	94.4	53	AE017226.1	Hypothetical protein [*Treponema* sp. OMZ 804]	774	91	100	75–848	WP_162662615.1
							Carbon–nitrogen hydrolase family protein [*Treponema denticola*]	894	98	100	998–1891	WP_187115772.1
Flip-MARS-11 (OL695869)	MARS2-MARS5	2890	Uncultured bacterium clone SSU24 gene cassette SSU24.1 genomic sequence	99	33	KT921490.1	Vicinal oxygen chelate (VOC) family protein [*Treponema denticola*]	393	100	100	68–460	WP_002683256.1
							Competence protein TfoX [uncultured bacterium]	222	100	100	629–850	ANC55521.1
							IS4 family transposase [*Treponema* sp.]	909	66	99	1630–2538	MBR1911588.1
IS431-5 (OL695870)	IS431-Inverse-F-IS431-inverse-R	1735	*Staphylococcus capitis* strain FDAARGOS_753 chromosome	91	100	CP053957.1	No significant similarity found	345	–	–	591–247	–
							Hypothetical protein [*Staphylococcus epidermidis*]	465	89	100	1299–835	MBE0334850.1
*tet*(M)-1 (OL695871)	*tet*(M)-inverse-F- *tet*(M)-inverse-R	5054	*Neisseria sicca* ATCC 29,256 chromosome	98	100	CP079820.1	Murein transglycosylase A [unclassified Neisseria]	1323	99	100	901–2223	WP_070606572.1
							Hypothetical protein	711	100	100	3456–2746	WP_070606574.1
							Holliday junction branch migration protein RuvA [unclassified *Neisseria*]	585	100	100	4342–3758	WP_070606583.1
*tet*(M)-2 (OL695872)	*tet*(M)-inverse-F- *tet*(M)-inverse-R	2456	*Streptococcus agalactiae* strain GBS6	99.6	85	CP007572.1	Hypothetical protein [*Streptococcus pneumoniae*]	195	98	100	901–1095	CDO19408.1
							Hypothetical protein AZK08_11845, partial [*Streptococcus pneumoniae*]	180	100	98.3	1629–1450	TXL69036.1
							IS21-like element helper ATPase IstB [*Streptococcus parasanguinis*]	315	100	39.8	2009–1695	WP_101770904.1
*tet*(M)-6 (OL695873)	*tet*(M)-inverse-F- *tet*(M)-inverse-R	4683	*Streptococcus pneumoniae* integrative and conjugative element ICESpnIC1, isolate 9611 + 04,103	100	100	HG799494.1	Hypothetical protein [*Streptococcus pneumoniae*]	195	98	100	901–1095	CDO19408.1
							Hypothetical protein HMPREF1885_00780 [*Streptococcus agalactiae*]	364	99	100	1815–1450	KXA59032.1
							23S rRNA (adenine(2058)-N(6))-methyltransferase Erm(B) [*Streptococcus*]	738	100	100	1916–2653	AYK27796.1
							Resolvase [*Streptococcus pneumoniae*]	555	99	100	3008–3562	VSD99176.1
							Tn*3* family transposase [*Enterococcus faecalis*]	671	99	74.8	3566–4236	EGO7700322.1
*tet*(M)-9 (OL695874)	*tet*(M)-inverse-F- *tet*(M)-inverse-R	2329	*Streptococcus pneumoniae* integrative and conjugative element ICE6BST90, isolate IC161	99.8	100	HG799499.1	Conjugal transfer protein [*Streptococcus agalactiae*]	186	95	100	910–1095	KAF1190941.1
							Helix-turn-helix transcriptional regulator [Bacteria] (orf9 Tn*916*)	354	100	100	1508–1155	WP_001227347.1
*xis-int*-9 (OL695875)	*xis-int*-inverse-F-*xis-int*-inverse-R	3704	*Streptococcus pneumoniae* strain 080,217 transposon Tn*6822*	90	100	MT489699.1	MFS transporter [*Streptococcus* sp. ZB199]	230	95	40.3	1134–905	QWL83132.1
							Cysteine-rich KTR domain-containing protein [Bacteria]	192	98	100	1258–1449	WP_001860868.1
							Helix-turn-helix transcriptional regulator [Bacteria] (orf9 Tn*916*)	354	100	100	1862–1509	WP_001227347.1
							Conserved hypothetical protein [*Streptococcus pneumoniae*] (orf10 Tn*916*)	147	100	100	1992–2138	ACA36714.1
							Sigma-70 family RNA polymerase sigma factor [*Streptococcus*] (orf7 Tn*916*)	423	99	100	2367–2789	WP_196313264.1
							Helix-turn-helix domain [Bacteria] (orf8 Tn*916*)	231	100	100	2786–3016	WP_000857133.1
							Hypothetical protein [Firmicutes] (orf5 Tn*916*)	252	100	100	3493–3242	WP_001845478.1

**Fig. 3:**
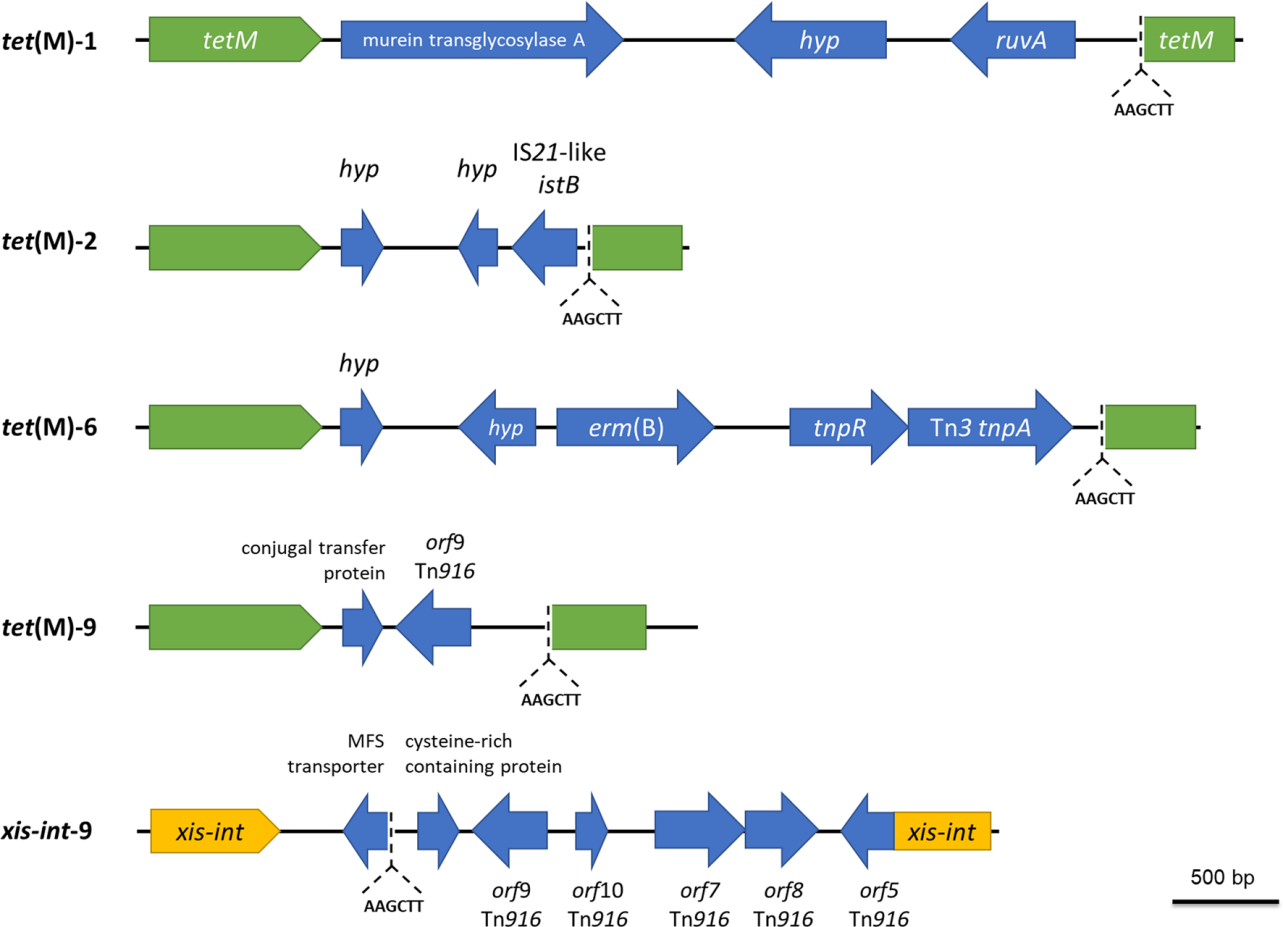
The predicted structures of the genetic context of Tn*916*-like elements detected from the oral metagenome by IPCR. Gene associated with Tn*916*-like elements (blue open arrows) were amplified outwards from either *tet*(M) (green open boxes) or *xis*-*int* (yellow open boxes). The location of HindIII restriction sites (AAGCTT) used in the self-circularization of the partial digested oral metagenome is indicated with dashed lines

The second group of samples was those amplified from a single conserved gene of MGEs on self-circularized DNA templates. It included the samples amplified by DNA primers targeting Tn*916*-family conjugative transposons: 4 *tet*(M) samples and 1 *xis*-*int* sample. (Fig. 3). HindIII restriction site, which was used for the self-circularization, was identified at the nucleotide position 230 of MFS transporter gene in *xis*-*int*-9 sample and the nucleotide position 63 of *tet*(M) gene of all *tet*(M) samples. *tet*(M)-6, *tet*(M)-9, and *xis*-*int*-9 were similar to integrative and conjugative elements ICESpnIC1, ICE6BST90 and Tn6822, respectively, which contained Tn*916*-related structures. For Tet(M)-2, it contained part of IS*21*-like helper ATPase, which was similar to the helper protein of IS*Cbe3* with 67% identities based on the BlastX analysis on ISFinder, while *tet*(M)-6 were shown to contain erythromycin resistance gene *erm(B*) next to a Tn3-family transposase and resolvase.

## Discussion

Mobile genetic elements play a crucial role in the antimicrobial resistance crisis as they can facilitate the movement of ARGs in the bacterial population. The surveillance of ARGs and other genes associated with MGEs is therefore important as they are highly likely to be disseminated, especially with selective pressure from all uses of antimicrobials. As the oral cavity is the gateway of the human body connecting to other organs like the gastrointestinal tract, MGEs could promote not only the movement of these genes from oral microflora to clinical pathogens but also have the potential to transfer their genes to other microbiomes as well. In our study, it is the first report that showed the uses of the IPCR-based approach to investigate the genetic contexts of MGEs in the oral metagenome, where several variants of Tn*916* conjugative transposons and novel integron genes cassettes were identified.

The uses of IPCR technique with metagenomic DNA has only been used previously to study the genetic context of resistance genes in the sediment metagenome, which was shown to be a cost-efficient method with higher sensitivity compared to the metagenomic sequencing approach [37]. With metagenomic sequencing, it requires the metagenome to have a deep enough sequencing depth to perform assembly properly, especially for the MGEs with low abundance, which increases the sequencing cost [3, 4]. Also, the structures of MGEs, which contain both conserved and variable regions, and multiple insertions of the same MGEs in bacterial genomes or bacterial cells in an environment increase the challenges for the assembly algorithms. IPCR could bypass these issues as the target DNA are enriched by the amplification of the low abundance MGEs, and the cloning of IPCR amplicons into vector allows us to use primer walking for sequencing, which are cheaper and the sequencing data can be analyzed through simple bioinformatic tools.

The detected integron GC and composite transposon samples were amplified from two DNA repeats of IS elements and *attC*, respectively, which could be amplified either from undigested linear or self-circularized oral metagenome. The amplification of genes between *attC* has been used commonly to investigate integron GCs in various environmental samples such as wastewater, soil, marine, and saliva [2, 14, 21, 56, 59]. All integron samples were related to *Treponema* species, corresponding to the design of MARS2 and MARS5 primers that was based on the *attC* sequence of the reverse integron of *T. denticola* ATCC 35,405 [9]. However, all samples detected by this primer pair in the previous studies contained single GC [56–59], but we identified 4 integron samples containing 2 GCs (including interspersed *attC* sites) in our study, which is the first time that double cassettes were recovered from the oral metagenome by PCR approach. It could occur from the additional gel extraction step to include only IPCR amplicons larger than 1 kb prior to the cloning step, which was not performed in the previous studies, in which the integron amplicons size from previous studies was 425–1263 bp, while the size of integron samples in this study was 1704–2890 bp.

Several PCR-based studies on integron in other environmental samples also recovered amplicons with multiple GCs previously marine sediment and deep-sea hydrothermal vent samples, where they found 44% and 32.5% of the samples to contain multiple GCs [13, 14]. This is possible as integrons can carry from a dozen of GCs in mobile integrons to hundreds of GCs in sedentary chromosomal integrons where each GC is separated by *attC* recombination sites (59–120 bp) with similar sequences to each other [17]. Therefore, multiple GCs can be recovered if DNA primers bind to *attC* that are not adjacent but bind to the *attC* located further instead.

Analyzing the detected integron GCs from the oral metagenome predicted several interesting proteins encoding by these GCs. Toxin-antitoxin proteins were detected in Flip-MARS-4 and Flip-MARS-5 GCs, same as in the previous studies using the MARS2-MARS5 primers [56–59]. The presence of toxin-antitoxin containing GCs is common for sedentary chromosomal integrons like *T. denticola* integrons as their function is to prevent random deletion of GCs and ensure the stability of the large GC arrays [19, 33, 48, 55]. Endonucleases were also predicted to be encoded by Flip-MARS-3 and Flip-MARS-4 GCs, which were also found in another study that performed an in silico analysis on metagenomic datasets of the Human Microbiome Project to identify integrons associated with *Treponema* spp [66]. The function of endonuclease enzymes in integrons has been suggested to involve in the cassette neoformation process [31]. Another gene, encoding VOC family protein, was also identified from Flip-MARS-11. Proteins in this family are metalloenzyme catalyzing diverse reactions through the formation of the partially closed beta-sheet barrel, which is formed by two βαβββ units, wrapping around the metal ions [20]. The important members of VOC family include glyoxalases I, fosfomycin resistance proteins and bleomycin resistance proteins [20, 27, 50].

Tn*916*-family conjugative transposons are one of the largest families of ICEs. They can be found either as a linear form in a bacterial genome or as a circular intermediate in which their backbones contain conjugative transfer, transcriptional regulation, and recombination modules. Many of the Tn*916* members also carry *tet*(M) as part of the accessory module. Tn*916*, the first and smallest member of this family, was isolated from *Enterococcus faecalis* DS16 [15], which later has been found in over 30 different bacterial genera [6, 43]. For the human oral cavity, Tn*916*-like elements have been found predominantly in oral streptococci, *Veillonella* spp., and *Neisseria* spp [29, 34, 42]. In our study, we used primers targeting *tet*(M) and *xis*-*int* genes on Tn*916*, which we identified 3 Tn*916*-like elements that were likely to derive from *Streptococcus pneumoniae* based on BlastN results.

The genes associated with the detected Tn*916* variants were including *erm(B)* erythromycin resistance gene, Tn3 family transposase and resolvase genes (*tnpA* and *tnpR*), and MFS transporter gene. The presence of *erm(B)* has been found in several members of the Tn*916* family, such as Tn*3872*, Tn*1545,* and Tn*6003* [8, 36]. However, the structure that *tnpA* and *tnpR* genes located downstream from *erm*(B) in *tet*(M)-6 sample was similar to a Tn*917* unit transposon structure, and the only transposon in the transposon registry that Tn*917* located next to *tet*(M) was Tn*3872* [36]. The presence of Tn*917* containing *erm*(B) on Tn*916*-family conjugative transposon represents the phenomenon where ARGs could be moved onto ICEs with the help of other non-conjugative elements, allowing these ARGs to be subsequently moved to other bacterial cells by the activity of ICEs. For the MFS transporter in the *xis-int*-9 sample, it is an efflux pump, which can be found in all phyla, from bacteria to mammals [39]. Their main function involves the uptake of sugars, but some are also involved in multidrug resistance (MDR), virulence, and biofilm formation in bacteria [40]. Examples of MFS transporter conferring MDR are LmrP protein in *Lactococcus lactis* conferring lincosamides, macrolides and streptogramins resistance, and MdfA protein in *E. coli* conferring chloramphenicol, erythromycin, ciprofloxacin and rifampicin resistance [10, 65].

MGEs were also identified in the detected MGEs, including IS*4*-family transposase in the Flip-MARS-11 sample and Tn*3*-family *tnpA*-*tnpR* in the *tet*(M)-6 sample. As the core structures of integrons do not have genes to catalyze their interchromosomal mobility, they have to rely on transposase or recombinase from other sources [16]. The IS4-family transposase detected in Flip-MARS-11 could be kept in an integron cassette array in the oral cavity for such a purpose as well. It is also the first time that IS element was found in a cassette array in the oral metagenome. The presence of transposase genes in cassette array was previously reported in *Xanthomonas* integrons [18]. For the *tet*(M)-2 sample, even though it was not amplified from Tn*916*-like elements, it contained the IS*21*-family helper protein next to the *tet*(M) gene. Therefore, there was a high probability for the *tet*(M) in this sample to be disseminated with the help of its associated IS*21*-family element, similar to the previous report that showed that IS*21-558* involved in the movement of *cfr* multidrug resistance gene [26].

Our study showed that the human oral microbiome is also one of the hot spots that contained a diverse pool of MGEs, which is consistent with other previous studies, where each MGE can be associated with various genes. For example, previous studies reported several different structures of IS*1216* composite transposons in the oral microbiome, containing *qrg* antiseptic resistance gene and universal stress protein A (*uspA)* gene [6, 56, 59]. Multiple Tn*916*-Tn*1545* family conjugative transposons were also identified from the oral bacteria, especially *Streptococcus* spp. such as Tn*6002* from *Streptococcus cristatus*, Tn*6815* from *Streptococcus mitis*, and Tn*6816* from *Streptococcus constellatus* [7, 34, 64], where their transferability between oral streptococci were also confirmed. As all uses of antimicrobials provide selective pressure for bacteria to develop and increase the transfer of these MGEs, the oral microbiome, as a major gateway to the whole body, have a high potential to act as a reservoir for the spread of antimicrobial resistance to other microbiomes through a myriad of MGEs [12]. Therefore, the oral microbiome is an important part that needs to be understood on a functional level for us to fully understand the antibiotic resistance crisis.

## Conclusion

To summarize, we have determined that genes associated with MGEs in the oral metagenome can be investigated through an IPCR-based approach, which requires less cost and simpler bioinformatic analysis compared to the metagenomic sequencing approach. As a result, several novel integron GCs, novel MGEs, and variants of Tn*916* were recovered from the oral metagenome.

## Supplementary Information

**Additional file 1:** Primers used in this study.

## Data Availability

The datasets used and/or analysed during the current study available from the corresponding author on reasonable request.

## Abbreviations

IPCR: Inverse PCR
EPS: Extracellular polysaccharides
ARGs: Antimicrobial resistance genes
HGT: Horizontal gene transfer
MGEs: Mobile genetic elements
ISs: Insertion sequences
GCs: Gene cassettes
