# Supplementary material for: Inverse PCR-based detection reveal novel mobile genetic elements and their associated genes in the human oral metagenome
Source: BMC Oral Health. 2022 May 27;22:210. doi: 10.1186/s12903-022-02209-y (PMC9137128; doi:10.1186/s12903-022-02209-y)
Supplement: Supplementary file 1 — Additional file 1: Primers used in this study. [file 12903_2022_2209_MOESM1_ESM.docx]

**Supplementary Table 1** Primers used in this study

| Primer name | Sequence (5’-3’) | Gene target | Reference |
| --- | --- | --- | --- |
| MGE confirmation primers | | | |
| IS*1*-F | GGTGCGTAACGGCAAAAG | IS*1* | This study |
| IS*1*-R | CCACCGATTTTGAGAACGAC | IS*1* | This study |
| IS*26*-F | AGCGTGACATCATTCTGTGG | IS*26* | This study |
| IS*26*-R | CCCAGGGGATCACCATAATA | IS*26* | This study |
| IS*431*-F | AAGGGAACGTGGTGTAAACG | IS*431* | This study |
| IS*431*-R | GCATGGCGAAAATCCGTAG | IS*431* | This study |
| IS*1182*-F | GCTCATGCCATTGTGCAGT | IS*1182* | This study |
| IS*1182*-R | ACCCGTGAAAGATGTCTTCG | IS*1182* | This study |
| IS*1216*-F | GCCGTGGGCTACTATCTTCG | IS*1216* | Tansirichaiya *et al.* (2016) |
| IS*1216*-R | CCCGAAGAGAGTGCCTTCTT | IS*1216* | Tansirichaiya *et al.* (2016) |
| IS*4351*-F | CAATGTCCCCCTTTCTCTCA | IS*4351* | This study |
| IS*4351*-R | GCCTGATAAGCCCGTTGG | IS*4351* | This study |
| IS*6100*-F | CGCTGGTATTGTCGCTATCC | IS*6100* | This study |
| IS*6100*-R | CCAATGCCAAAAGCTCTCTC | IS*6100* | This study |
| IS*Aba1*-F | GTGCTTTGCGCTCATCAT | IS*Aba1* | This study |
| IS*Aba1*-R | CCTATCAGGGTTCTGCCTTC | IS*Aba1* | This study |
| IS*26*-F | AGCGTGACATCATTCTGTGG | IS*26* | This study |
| IS*26*-R | CCCAGGGGATCACCATAATA | IS*26* | This study |
| IS*1999*-F | CGCCAGCAATTCTTTCTCC | IS*1999* | This study |
| IS*1999*-R | CCTCTAAGGCGCTGATTGAA | IS*1999* | This study |
| IS*30*-F | GAAGAACGGAACAGGCTTCA | IS*30* | This study |
| IS*30*-R | TTAGCTGAGCAGCAACCAGA | IS*30* | This study |
| IS*16*-F | GAAAGCGGTGCGAATGATAC | IS*16* | This study |
| IS*16*-R | GCCATGCCTTTCTTTCTGAG | IS*16* | This study |
| IS*256*-F | CCCAGGAGGACTTTTACATGAC | IS*256* | This study |
| IS*256*-R | TTGGAGCCGTTCTTATGGAC | IS*256* | This study |
| IS*630*-F | TGACGAACGACGCCTGAT | IS*630* | This study |
| IS*630*-R | AGCTGCACTGATGATTACGC | IS*630* | This study |
| IS*1071*-F | CCCACTTTTGAGGTTCACGA | IS*1071* | This study |
| IS*1071*-R | CGGAGGTGGTGTTGGTAGAT | IS*1071* | This study |
| IS*4351*-F | CAATGTCCCCCTTTCTCTCA | IS*4351* | This study |
| IS*4351*-R | GCCTGATAAGCCCGTTGG | IS*4351* | This study |
| IS*Aba26*-F | GTCCATTTCTTCGCAGCAT | IS*Aba26* | This study |
| IS*Aba26*-R | CCGAATTGGCTAAAGGTC | IS*Aba26* | This study |
| IS*Pa12*-F | ATTGAACGCTTTCGCCTGAT | IS*Pa12* | This study |
| IS*Pa12*-R | CCCTGAACAAGCCCATTTTA | IS*Pa12* | This study |
| IS*Pa13*-F | GGTTAAAATGGCTGCTCCTG | IS*Pa13* | This study |
| IS*Pa13*-R | CGCCACTTATTTGGTCTCGT | IS*Pa13* | This study |
| IncP-1α-F | ATGACGACCAAGAAGCG | IncP-1α | Götz *et al.* (1996) |
| IncP-1α-R | AACCCCCAGCCGGAACTG | IncP-1α | Götz *et al.* (1996) |
| IncP-9-F | CCAGCGCGGTACWTGGG | IncP-9 | Rainey (1999) |
| IncP-9-R | GTCGGCAICTGCTTGAGCTT | IncP-9 | Rainey (1999) |
| IncQ-F | CTCCCGTACTAACTGTCACG | IncQ | Götz *et al.* (1996) |
| IncQ-R | ATCGACCGAGACAGGCCCTGC | IncQ | Götz *et al.* (1996) |
| IncP-1β-F | CGAAATTCRTRTGGGAGAAGTA | *IncP-1β* | Götz *et al.* (1996) |
| IncP-1β-R | CGYTTGCAATGCACCAGGTC | *IncP-1β* | Götz *et al.* (1996) |
| *tnpA*-F | TGCGCTCCGGCGACATCTGG | *tnpA* | Kiiru *et al.* (2013) |
| *tnpA*-R | TCAGCCCGGCATGCACGCG | *tnpA* | Kiiru *et al.* (2013) |
| *tnp*R-F | GTTCAGCRCTTCGACCAG | *tnpR* | Pearson *et al.* (1996) |
| *tnp*R-R | YRAGGGTTYCMGCTGAT | *tnpR* | Pearson *et al.* (1996) |
| *tet*(M)-F | GTRAYGAACTTTACCGAATC | *tet*(M) | Guardabassi *et al.* (2000) |
| *tet*(M)-R | ATCGYAGAAGCGGRTCAC | *tet*(M) | Guardabassi *et al.* (2000) |
| *xis*-*int*-F | CGCCAAAGGGTCTTGTATATG | *xis-int* of Tn*916* | Marra & Scott (1999) |
| *xis*-*int*-R | GCTGTAGGTTTTATCAGCTTTTGC | *xis-int* of Tn*916* | Marra & Scott (1999) |
| Flip-MARS2 | GCTTCAACCTGACATTGC | Integron *attC* recombination site | This study |
| Flip-MARS5 | CGCTTAACCTGCATTTGCG | Integron *attC* recombination site | This study |
| Inverse PCR primers | | | |
| IS*1*-inverse-F | GTCGTTCTCAAAATCGGTGG | Genetic context of IS*1* | This study |
| IS*1*-inverse-R | TTTTGCCGTTACGCACC | Genetic context of IS*1* | This study |
| IS*26*-inverse-F | TATTATGGTGATCCCCTGGG | Genetic context of IS*26* | This study |
| IS*26*-inverse-R | CCACAGAATGATGTCACGCT | Genetic context of IS*26* | This study |
| IS*1182*-inverse-F | CGAAGACATCTTTCACGGGT | Genetic context of IS*1182* | This study |
| IS*1182*-inverse-R | ACTGCACAATGGCATGAGC | Genetic context of IS*1182* | This study |
| IS*1216*-inverse-F | AAGAAGGCACTCTCTTCGGG | Genetic context of IS*1216* | This study |
| IS*1216*-inverse-R | CGAAGATAGTAGCCCACGGC | Genetic context of IS*1216* | This study |
| IS*4351*-inverse-F | CCAACGGGCTTATCAGGC | Genetic context of IS*4351* | This study |
| IS*4351*-inverse-R | TGAGAGAAAGGGGGACATTG | Genetic context of IS*4351* | This study |
| IS*6100*-inverse-F | GAGAGAGCTTTTGGCATTGG | Genetic context of IS*6100* | This study |
| IS*6100*-inverse-R | GGATAGCGACAATACCAGCG | Genetic context of IS*6100* | This study |
| IS*Aba1*-inverse-F | GAAGGCAGAACCCTGATAGG | Genetic context of IS*Aba1* | This study |
| IS*Aba1*-inverse-R | ATGATGAGCGCAAAGCAC | Genetic context of IS*Aba1* | This study |
| IS*431*-inverse-F | AAGGGAACGTGGTGTAAACG | Genetic context of IS*431* | This study |
| IS*431*-inverse-R | GCATGGCGAAAATCCGTAG | Genetic context of IS*431* | This study |
| IncP-*1*α-inverse-F | CAGTTCCGGCTGGGGGTT | Genetic context of IncP-*1*α | This study |
| IncP-*1*α-inverse-R | CGCTTCTTGGTCGTCAT | Genetic context of IncP-*1*α | This study |
| IncP-9-inverse -F | AAGCTCAAGCAGCTGCCGAC | Genetic context of IncP-9 | This study |
| IncP-9-inverse -R | CCCAWGTACCGCGCTGG | Genetic context of IncP-9 | This study |
| IncP-1β-inverse -F | ATCAGCKGRAACCCTYR | Genetic context of IncP-1β | This study |
| IncP-1β-inverse -R | TACTTCTCCCAYAYGAATTTCG | Genetic context of IncP-1β | This study |
| *tnpA*-inverse-F | CGCGTGCATGCCGGGCTGA | Genetic context of *tnpA* | This study |
| *tnpA*-inverse-R | CCAGATGTCGCCGGAGCGCA | Genetic context of *tnpA* | This study |
| MARS5 | GCAATGTCAGGTTGAAGC | Genetic context of integron *attC* | Tansirichaiya *et al.* (2016) |
| MARS2 | CGCAAATGCAGGTTAAGCG | Genetic context of integron *attC* | Tansirichaiya *et al.* (2016) |
| *tet*(M)-inverse-F | GATTCGGTAAAGTTCRTYAC | Genetic context of *tet*(M) | This study |
| *tet*(M)-inverse-R | GTGAYCCGCTTCTRCGAT | Genetic context of *tet*(M) | This study |
| *xis-int*-inverseF | CGCCAAAGGGTCTTGTATATG | Genetic context of *xis-int* | This study |
| *xis-int*-inverse-R | GCTGTAGGTTTTATCAGCTTTGC | Genetic context of *xis-int* | This study |
| Primers for sequencing | | | |
| T3 | ATTAACCCTCACTAAAGGGA | T3 forward sequencing | Genewiz |
| T7 | TAATACGACTCACTATAGGG | T7 reverse sequencing | Genewiz |
| M13F | GTAAAACGACGGCCAG | M13 forward sequencing | Genewiz |
| M13R | CAGGAAACAGCTATGAC | M13 reverse sequencing | Genewiz |
| Flip-MARS-3-F1 | TGGAAAAACGTGACGCAGTA | Flip-MARS-3 | This study |
| Flip-MARS-3-R1 | CGCTAAGGGAATTGACGAAA | Flip-MARS-3 | This study |
| Flip-MARS-4-F1 | GAAATGTCCTGTTGCGGAGT | Flip-MARS-4 | This study |
| Flip-MARS-4-R1 | GCCAAAGGCACATCAACCTA | Flip-MARS-4 | This study |
| Flip-MARS-11-F1 | TTCGACAAAGGGTACTGCAA | Flip-MARS-11 | This study |
| Flip-MARS-11-R1 | ACTGCCGGAACAAAAGAGAA | Flip-MARS-11 | This study |
| IS*431*-5-F1 | CAGTCTTTTGTTGCCCTGAA | IS*431*-5 | This study |
| IS*431*-5-R1 | ATACCTGTCCGCCTTTCTCC | IS*431*-5 | This study |
| *tet*(M)-1-F1 | CATTACGCTCGTCAGCATTG | *tet*(M)-1 | This study |
| *tet*(M)-1-F2 | CAGATGATCCGGTCGAACTT | *tet*(M)-1 | This study |
| *tet*(M)-1-F3 | CGGCAAAATGAAAACTACCG | *tet*(M)-1 | This study |
| *tet*(M)-1-R2 | CCTTCCGTCAACTGGTCAAA | *tet*(M)-1 | This study |
| *tet*(M)-1-R2 | GAAGGCGAAAAACTGCTGAC | *tet*(M)-1 | This study |
| *tet*(M)-1-R3 | GGCAAAACAGGTAAGGGTGA | *tet*(M)-1 | This study |
| *tet*(M)-2-F1 | CCATACGCAAGACCAATCAC | *tet*(M)-2 | This study |
| *tet*(M)-2-R1 | TGGATATTGTGTCCTGTATGTGG | *tet*(M)-2 | This study |
| *tet*(M)-6-F1 | GGATACCGGGCCAAACAAAG | *tet*(M)-6 | This study |
| *tet*(M)-6-F2 | GCTCCTTGGAAGCTGTCAGT | *tet*(M)-6 | This study |
| *tet*(M)-6-R1 | CAGGAGTGATTACATGAAC | *tet*(M)-6 | This study |
| *tet*(M)-9-F1 | TGGATATTGTGTCCTGTATGTGG | *tet*(M)-9 | This study |
| *tet*(M)-9-R1 | CGTGAACAAGTGGGAGCATT | *tet*(M)-9 | This study |
| *xis-int*-9-F1 | CCACTGGTTCTATGCCGATA | *xis-int*-9 | This study |
| *xis-int*-9-F2 | GTACTTCATGGCGACGTTGA | *xis-int*-9 | This study |
| *xis-int*-9-R1 | TGCAAAGCAGAGAGAGTTCGT | *xis-int*-9 | This study |
| *xis-int*-9-R2 | CTGTAATCGTCGGGTGCTTT | *xis-int*-9 | This study |
